# Mouse microbiomes: overlooked culprits of experimental variability

**DOI:** 10.1186/s13059-019-1723-2

**Published:** 2019-05-29

**Authors:** Maria-Luisa Alegre

**Affiliations:** Department of Medicine, Section of Rheumatology, University of Chicago, South Maryland Avenue, Chicago, IL 60637 USA

## Abstract

Recent studies have found differences between almost genetically identical mice that were attributable to differences between their microbiomes. One example of mice from different vendors having different susceptibilities to infection by *Salmonella enterica* serovar Typhimurium has just been published in *Nature Microbiology*.

Genetic differences between individuals are major drivers of phenotypic variations, which include differences in physical traits, susceptibility to disease, or responsiveness to therapies. More recently, environmental factors have also been implicated in promoting phenotypic variability. In particular, differences in the colonizing microbiota are often suspected or blamed for lack of reproducibility when different experiments or research centers produce inconsistent data. For instance, completely different results were obtained by each of four American research laboratories in a pre-clinical consortium that used mice of the same genotype to investigate, in parallel, whether anti-CD3 and IL-1 blockade therapy could prevent the onset of type 1 diabetes (as had been previously reported) [1]. Even when the responsibility of the microbiota has been confirmed, there is rarely a proven link between a variable phenotype and the causality of specific microbial strains and a definition of their mechanism of action. A recent study published in *Nature Microbiology* identifies both specific commensals and the mechanism underlying different susceptibility to *Salmonella* Typhimurium infection in mouse strains of similar genotypes [2]. Velazquez et al. [2] show that mice with increased susceptibility to *S.* Typhimurium infection and lethality lack low-abundance bacteria of the *Enterobacteriaceae* family. They also show that, in resistant mice, these *Enterobacteriaceae* bacteria can bloom during *S.* Typhimurium infection and compete with the pathogen for resources that are essential for the expansion of the *S.* Typhimurium population.

The authors compared genetically similar mice (C57BL/6) obtained from different vendors and found that, although all mice succumbed to a high-dose challenge with *S.* Typhimurium, only mice obtained from Jackson Laboratories (Jax) were susceptible to colonization and lethality by low doses of *S.* Typhimurium. These differences resulted from variation in gut microbiota, as fecal microbiota transfer from the different mice into germ-free Swiss Webster mice recapitulated the susceptibility or resistance to *S.* Typhimurium of the fecal donor in the Swiss Webster mice. Co-housing of Jax mice with mice from the other vendors only conferred resistance to *S.* Typhimurium in a fraction of Jax mice, so the authors hypothesized that resistance to *S.* Typhimurium might be driven by minority species present in the intestine of non-Jax mice that may be less easily transferred than high-abundance species. Fecal microbiota sequencing of the 16S rRNA gene and linear discriminant analysis, comparing microbiota from Jax mice before co-housing with those from post-co-housing-susceptible and from post-co-housing-resistant Jax mice, revealed increased abundance of *Enterobacteriaceae* in resistant mice. Indeed, only the fecal samples of resistant mice contained *Enterobacteriaceae* that were culturable on MacConkey agar. Furthermore, the transfer of bulk-cultured bacteria or of individual colonies, including *Escherichia coli*, tended to confer resistance to *S.* Typhimurium challenge. These *Enterobacteriaceae* inhibited the growth of *S.* Typhimurium in aerobic but not anaerobic culture conditions in vitro, suggesting that *Enterobacteriaceae*, which are facultative anaerobes, use their respiratory metabolism to compete with *S.* Typhimurium for resources. Indeed, transfer into Jax mice of wild-type *E. coli* conferred protection against *S.* Typhimurium, whereas transfer of engineered mutants that were deficient for aerobic respiration did not. Velazquez et al. [2] propose that inflammation induced by *S.* Typhimurium infection results in a disruption of gut homeostasis and a reduction in the natural anaerobic status of the colon. The increased bioavailability of oxygen enables the expansion of the *Enterobacteriaceae*, if they are present at steady state, which then compete with *S.* Typhimurium for critical resources and confer some level of protection against infection to the mice that carry them. Thus, Velazquez et al. [2] have identified a family of low-abundance intestinal bacteria that are responsible for phenotypic variation in susceptibility to *S.* Typhimurium pathogenic infection. Jax mice were also recently reported to be more susceptible than Taconic Farms (Tac) mice to gastric infection by *Helicobacter pylori*, though whether the distinct stomach microbiota observed in Jax and Tac mice are responsible for this phenotypic difference remains to be demonstrated [3].

The approach of comparing mice of similar genotypes obtained from different vendors and observing distinct phenotypes was pioneered by Ivanov and colleagues [4]. They discovered that C57BL/6 mice from Tac but not from Jax harbored a particular population of interleukin-17-producing intestinal T cells, termed Th17 cells, and that fecal transfer from Tac to Jax mice restored this Th17 population in Jax mice. This difference was due to the presence of a segmented filamentous bacterium in the terminal ileum of Tac but not Jax mice, and the introduction of this bacterium into Jax mice was sufficient to confer the Th17 phenotype. Thus, differences in microbiota between animals of the same genotype can instruct immune differences even at steady state, and this immune modulation can dramatically alter susceptibility to a variety of diseases from infections to autoimmune conditions.

Many other examples of phenotypic variations that are ascribed to variations in gut microbiota have since been discovered by comparing Jax and Tac C57BL/6 mice. Many reports invoke a microbiota-dependent immune impact as an intermediary between microbiota variability and phenotypic differences. For instance, Jax mice were shown to control the growth of a melanoma tumor better than Tac mice and to have superior responses to an anti-tumor immunotherapy [5]. These differences were due to the presence in Jax mice of intestinal bacteria of the *Bifidobacterium* genus, and fecal transfer from Jax to Tac mice or oral administration into Tac mice of a mixture of *Bifidobacterium* strains improved tumor control and susceptibility to immunotherapy by Tac mice. In this example, gut *Bifidobacterium* species modulated the immune system of the host such that it could better eliminate the tumor.

In a skin transplantation model, Jax mice displayed slower skin graft rejection than Tac mice [6]. Here the phenotypic difference was ascribed to the presence of the genus *Alistipes* in the intestine of Jax mice. The transfer of *Alistipes* to Tac mice by co-housing or through fecal gavage correlated with slower kinetics of skin-graft rejection, demonstrating the dominance of one microbial genotype on the graft-rejection phenotype. Why microbiota-dependent modulation of the immune system in Jax mice would confer better tumor rejection but slower transplant rejection remains to be teased out.

Individual variation in the microbiota is not only a source of experimental variability in laboratory mice but probably also a source of susceptibility to disease, to therapeutic success, or to drug side effects in humans. Indeed, the composition of the intestinal microbiota has been causally linked to susceptibility or resistance to cow milk allergy in infants [7] and to responsiveness to immunotherapy in cancer patients [8]. Moreover, the presence of gut commensals that have β-glucuronidase activity is known to drive diarrhea after treatment with irinothecan, an anticancer drug, by activating the metabolic toxicity of the drug [9]. In fact, intestinal levels of β-glucuronidase might serve as a biomarker to predict this toxicity.

Together, all of these studies reveal at least two main pathways by which the microbiota might affect various phenotypes (Fig. 1). One, as suggested by Velazquez et al. [2] and by studies on irinotecan, implies that intestinal commensals have direct effects on gut pathogens or drugs, without any involvement of an immune phenotype intermediary. The other pathway, as demonstrated by the additional studies highlighted here, depends on an immune phenotype that is associated with an individual’s microbiota composition. Stappenbeck and Virgin [10] have proposed that individuals should be thought of as the metagenomic sum of the host genome and the microbiome genome, as certain phenotypes are conferred by host genetics, others by the microbiome, and yet others by an interaction between both. Thus, when studying the role of host genotype on phenotype, it is important to take into consideration the genotype of the colonizing microbiota. Use of co-housed mutant and control littermates can minimize variation between groups not only in background genes but also in the microbiota, though, as demonstrated by Velazquez et al. [2], low-abundance bacteria may not be equally disseminated by co-housing. In addition, pre-clinical researchers should carefully record and report the source of the animals, type of diet, water treatment, light cycle, or other variables that may affect the microbiota, to improve the likelihood that their data can be reproduced by other investigators. Finally, translational or clinical researchers might be increasingly required to factor microbiome make up as an additional dimension for personalized medicine.

**Fig. 1:**
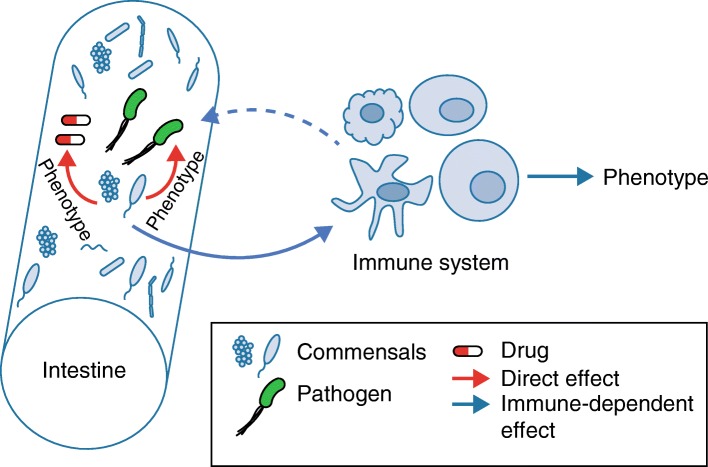
Interpersonal variations in microbial composition can have a major impact on phenotypic readouts. The intestinal microbiota can modulate diverse phenotypes from susceptibility to disease, to responsiveness to treatment or predisposition to drug side effects, either directly (*red arrows*) or by modifying the immune system (*blue arrows*)
